# Secondary Analysis of a Brief Parent-Implemented NDBI on Activity-Engaged Triadic Interactions Within Mother–Child Dyads

**DOI:** 10.3390/bs16010147

**Published:** 2026-01-20

**Authors:** Ciara Ousley, Tess Szydlik, Shelby Neiman, Nyah Elliott

**Affiliations:** Department of Special Education and Communication Disorders, University of Nebraska–Lincoln, Lincoln, NE 68583, USA

**Keywords:** autism, naturalistic developmental behavioral intervention, triadic interactions, parent-implemented intervention, telepractice

## Abstract

Family-implemented interventions are evidence-based practices used to support a range of developmental outcomes, including social communication. Social communication is a broad construct that encompasses a variety of skills, from foundational abilities such as joint attention (i.e., two people attending to the same object or event) to more advanced behaviors like triadic interactions (i.e., responding to or initiating conversation that involves reciprocal interactions). In a previous study, we examined the effects of a brief, parent-implemented Naturalistic Developmental Behavioral Intervention (NDBI), delivered over telepractice with video feedback coaching. The intervention resulted in increased strategy use by all mothers and the frequency of communication for three young children. In the current study, we conducted a secondary analysis of those data to explore whether the communication-focused intervention produced a collateral effect on activity-engaged triadic interactions (i.e., mother–child–mother or child–mother–child exchanges while simultaneously engaging in a joint activity). Although a functional relation was not established, critical theoretical implications are posed. These findings highlight the need for future research to break apart complex skills into subskills to detect any subtle changes in child outcomes. Limitations and directions for future research are discussed.

## 1. Introduction

Family-implemented interventions are evidence-based practices for young children with autism and other developmental disabilities, supporting a variety of developmental outcomes, such as social communication (Hume et al., 2021). Social communication is an umbrella term that includes a variety of skills, ranging from early developing skills, such as joint attention (i.e., two people drawing attention to the same object/event), to advanced skills like social referencing (i.e., socially seeking information from another person after an event). Typically, children gain these social communication skills in a similar sequence, each building upon the other and becoming more complex (Bakeman & Adamson, 1984). Thus, there is a general understanding that to build more complex skills, we must target foundational skills that constitute the more complex skill. Two skills that are quite complex to teach young children with autism and developmental delays, due to their reliance on multiple language and social communication skills, are triadic interactions and joint engagement.

Joint engagement, or periods of time in which two individuals actively focus on a shared object or event, is a complex skill that has implications for downstream language skills (Shih et al., 2021). For example, joint engagement comprises reciprocal interactions between two individuals, including turn-taking and imitation (Hansen et al., 2018). Within reciprocal interactions falls the skill of triadic interactions, in which two parties have a series of back-and-forth shared interactions (e.g., mother–child–mother or child–mother–child communication exchanges). This skill can be further complicated when requiring the reciprocal interaction to occur while being jointly engaged with an object or activity (i.e., activity-engaged triadic interactions). A key factor in building early language and communication skills for young children, particularly those who are autistic or have developmental delays, is parents’ modeling and presentation of language within direct, reciprocal interactions that shape subsequent child language development (Fusaroli et al., 2019).

### 1.1. Early Language Interventions for Young Children with Autism and Developmental Delays

Early interventions that involve family members supporting the language and communication skills of young children with delays can greatly improve outcomes in cognitive development (McIntyre et al., 2021; Wallander et al., 2014) and language skills (Belanger et al., 2021), particularly spoken language (Fuller & Kaiser, 2020; Hampton & Kaiser, 2016). Due to the complexity of triadic interactions (i.e., responding to a communication partner or initiating interaction with a communication partner and responding to the communication partner), early interventions that focus on pre-requisites of triadic interactions (e.g., total communication, joint attention) are critical for child success, particularly in expressive and receptive language development (Gulsrud et al., 2014; Lee et al., 1998; Shih et al., 2021). Targeting joint attention and interaction skills during early intervention has demonstrated increases in expressive language years later (Gulsrud et al., 2014).

Naturalistic developmental behavioral interventions (NDBIs) are an approach to early intervention that can lead to successful gains in a variety of social communication skills (Sandbank et al., 2020; Schreibman et al., 2015). NDBIs incorporate applied behavior analytic principles to teach developmentally appropriate skills in natural environments, often including play (Schreibman et al., 2015). One of the key foci of NDBIs is aligning interventions for young children with autism and other developmental disabilities with typical developmental trajectories (Ousley et al., 2025). Through regular and proficient parent implementation of NDBI strategies, there has been demonstrated improvement in child social communication, language, and joint attention, which are skills involved in triadic interactions and joint engagement (Hampton et al., 2021; Rakap & Rakap, 2014; Roberts & Kaiser, 2015).

### 1.2. Current Study

Ousley et al. (2022) recently evaluated the effects of a parent-implemented NDBI using video feedback coaching delivered via telepractice on parent strategy use and child social communication skills. Using a concurrent multiple baseline single-case experimental design employed across three parent–child dyads, parents received a 1–1.5 h training via telepractice on five NDBI strategies (i.e., Model Language, Arrange Environment, Follow and Imitate the Child, Provide Wait Time, and Reward and Expand Child Communication) followed by weekly individualized video feedback coaching sessions and joint reflections with a researcher. The primary dependent variable was the percentage of 10 s intervals with a parent-implemented strategy, and the secondary dependent variable was child communication. All children had diagnosed disabilities or delays (i.e., autism spectrum disorder, Williams syndrome, developmental delay) and ranged from 2 to 5 years of age. Parents met with the researcher via Zoom to record 10 min play sessions, where the researcher turned off their camera and muted themselves, while the parent played with the child within their home using their typical toys and activities. Upon visual analysis, there was a strong functional relation between the intervention and parent strategy use. Parents used all five strategies consistently throughout the intervention. Two of the three young children increased the frequency of their communication target. The parents provided high ratings on a social validity interview.

In the current study, we conducted a secondary analysis of the raw data from Ousley et al. (2022) to understand if there was a collateral effect on a more complex skill that integrated the primary (parent strategy use) and secondary (child communication) dependent variables in one variable. In other words, we were curious if the parents were able to string together their strategies by following the child’s lead and interests, providing antecedent strategies to prompt communication, allowing the child to communicate, and responding to the child’s communication attempt, all while remaining engaged in play. Thus, we developed the definition of activity-engaged triadic interactions: triadic interactions (i.e., mother–child–mother or child–mother–child communicative exchanges) while simultaneously remaining jointly engaged in an activity together. This skill required both the parent and child to maintain high levels of engagement and implementation, each of which was demonstrated to be successful (in isolation) in the original study (Ousley et al., 2022). Secondary analyses of data occur when new analyses are undertaken for existing data. These occur for a variety of reasons, including posing alternative research questions and examining different dependent variables (Lombardi et al., 2023). Secondary analyses of single-case experimental design data have been successful in identifying additional outcomes from a completed study involving participants with developmental conditions (Bhana Lopez et al., 2024; Bourque & Goldstein, 2020), thus increasing the practical understanding and impact an intervention can have. Our specific research question was as follows: Is there a collateral functional relation following a telepractice parent-implemented NDBI with video feedback coaching and joint reflections on an increase in activity-engaged triadic interactions between mother–child dyads?

## 2. Methods

Following Institutional Review Board (IRB) approval, a secondary analysis of raw video data from a concurrent single-case multiple baseline design employed across three parent–child dyads was evaluated on the percentage of intervals with activity-engaged triadic interactions (Ousley et al., 2022). Ten-minute videos were coded using 10 s partial interval recording. All participants provided consent for the video recordings to be used for additional analyses, following IRB-approved procedures.

### 2.1. Participants and Setting

English-speaking parent–child dyads were recruited across the United States to participate. Parents had to be 18 years or older and the primary caregiver of the child. The child had to be between the ages of 2 and 5 years of age, have normal or corrected hearing, have a communication domain score at least 1 standard deviation below the mean of the Vineland Adaptive Behavior Scale—Third Edition (VABS-3; Sparrow et al., 2016), and have an official diagnosis that qualified the child for services under the Individuals with Disabilities Education Act (Individuals with Disabilities Education Improvement Act of 2004, Pub. L. No. 108–446, § 1400 et seq, 2004) per parent report. Participants (i.e., parent–child dyads) engaged in play activities in their homes. See below and Table 1 for additional participant information.

Three parent–child dyads were qualified, and all sessions took place synchronously (i.e., the researcher logged into Zoom to observe a 10 min play session) via Zoom. All names are pseudonyms to protect confidentiality. Johnny was a 5-year-2-month-old white male with autism spectrum disorder. His expressive communication skills were age equivalent to 1 year 11 months of age, and his play and leisure skills were age equivalent to 2 years 2 months of age according to VABS-3. His mother, Rose, was white and served as the interventionist. Johnny resided with his mother and father in a rural Mid-Atlantic portion of the United States. Sessions for Johnny and Rose included playing with figurines (e.g., Peppa Pig) and toy sets (e.g., school building). Cece was a 2-year-2-month-old white female with a medical diagnosis of Williams syndrome and a developmental delay in language. Her expressive communication skills were age equivalent to 11 months of age, and her play and leisure skills were equivalent to a 1 year 11 months of age according to VABS-3. Her mother, Pam, was white and served as the interventionist. Cece resided with her mother and father in a suburban Northeastern area of the United States. Sessions for Cece and Pam included dancing to music, reading books, and playing on a slide. Ron was a 2-year-0-month-old white male with a developmental delay in communication. His expressive communication skills were age equivalent to one year 5 months of age, and his play and leisure skills were age equivalent to one year 11 months of age according to VABS-3. His mother, Leslie, was white and served as the interventionist. Ron resided in a rural Midwestern section of the United States. Sessions for Ron and Leslie included sensory items (e.g., kinetic sand, rice in bin), toy cars, and figurines.

### 2.2. Independent and Dependent Variables

The independent variable is described in detail in Ousley et al. (2022). To summarize, the first author provided a brief (1–1.5 h) one-time training individually to each of the three mothers and individualized video feedback each week. The training was on five NDBI strategies (i.e., Model Language, Arrange Environment, Follow and Imitate the Child, Provide Wait Time, and Reward and Expand Child Communication) via Zoom using a PowerPoint. The training followed a similar structure across participants but included individualization to each dyad (e.g., examples and screenshots from baseline). Each training, the researcher and mother (a) reviewed importance of communication; (b) analyzed an interaction from baseline highlighting the antecedent–behavior–consequence; (c) co-developed a learning target or goal for the child; (d) applied each strategy to their child’s interests; and (e) engaged in a scenario-based discussion on a hypothetical scenario and how the strategies could be used with the toys they had in the home. Following the training, the researcher developed and shared video feedback coaching with each parent, highlighting the previous week’s interactions from the recorded sessions. Coaching occurred for 4–6 weeks. Video feedback averaged 3 min 10 s and included four short video clips: three highlighting the parent successfully using the strategies and one emphasizing one strategy to focus on for the week (which highlighted the parent accurately implementing that strategy to build self-confidence). The strategy of focus was based on clinical judgment and rotated each week as the need arose.

The primary dependent variable for the current study was the percentage of intervals with activity-engaged triadic interactions. Secondarily, we coded each interval in a ranking system between unengaged, onlooker, person engaged, and object engaged. Specifically, we used 10 s partial interval recording as the measurement system. While partial interval recording can often overestimate the duration of behavior, interval recording is appropriate when there is an unclear beginning and ending to episodes. With activity-engaged triadic interactions having various combinations and interaction length (e.g., mother–child–mother or child–mother–child–mother–child), we used partial interval recording. This aligns with previously published research evaluating similar dependent variables (Shih et al., 2021; Vernon et al., 2012). We manually coded each interval based on the highest level demonstrated in the interval. The third and fourth authors served as coders. See Table 2 for rankings and operational definitions, and Section 2.4 below for specific coding procedures.

### 2.3. Procedures

Two phases of intervention occurred: baseline and intervention. Prior to baseline but after consenting to the study, the researcher met with the parent twice via Zoom to conduct assessments (e.g., demographic questionnaire, researcher-created preference assessment, VABS-3). All sessions in both phases were recorded via synchronous Zoom meetings. Each phase is briefly described below.

#### 2.3.1. Baseline

During baseline, the researcher and parent logged into a secure Zoom room and greeted one another. Once the parent was ready to begin the session, the researcher verified that the technology was working, requested the parent to play with their child for 10 min while remaining in the video frame, and asked if there were any questions. After answering any questions, the researcher turned off her camera, muted her microphone, and set a 10 min timer. At the end of the timer, the researcher turned on her audio and camera. No feedback or coaching was provided at any point during the meeting.

#### 2.3.2. Intervention

During the intervention, the researcher and parent logged into a secure Zoom room and greeted one another. The researcher then shared video feedback from the previous week with the parent, reviewed the five strategies, and asked if there were any questions. The researcher answered any questions the parent had, verified that the technology was working, requested the parent to play with their child for 10 min while remaining in the video frame, and reminded the parent to engage in the five strategies they learned in the training and reviewed in the video feedback. Then, the researcher turned off her video and muted herself while observing the play time interaction between the parent and child. After 10 min, the researcher turned on her camera and engaged in joint reflections with the parent about the interaction (e.g., asking the mother, “how do you think that went?”, then reassuring their perceptions if positive or reframing parent responses to highlight strong interactions or strategy use if negative, or providing any requested feedback). The child was often occupied during joint reflections (e.g., with another caregiver, provided a snack or toy) so the coach and parent could converse about the session.

### 2.4. Data Collection and Analysis

All 10 min play sessions were video recorded and broken into 10 s intervals based on video timestamps. The first author trained the third and fourth authors (female undergraduate researchers studying special education or speech–language pathology) on coding engagement levels (i.e., unengaged, onlooking, object engaged, person engaged) and activity-engaged triadic interactions (i.e., mother–child–mother or child–mother–child exchanges while simultaneously being engaged in a shared activity). The third and fourth authors watched the 10 min videos and manually coded each 10 s interval using partial interval recording to identify the highest level of engagement displayed by the dyad in each interval. See Table 2 above for ranking and operational definitions. Interobserver agreement (IOA) data were collected on at least 30% of both phases (i.e., baseline and intervention) for all participants using interval-by-interval comparisons. Any disagreements were discussed between coders (i.e., third and fourth authors). If the two coders could not come to a consensus for an interval, the coders and the first author reviewed the interval, reviewed definitions, and decided on the correct code for the interval. These conversations included all coders to ensure there was consensus to inform future coding. IOA was 95% (87–100%), 95% (85–100%), and 94% (90–97%) for each dyad, respectively.

Graphical data were analyzed using two methods: visual analysis (i.e., changes in level, trend, variability, immediacy of effects, and consistency) and between-case standardized mean difference effect size (i.e., scdhlm using a web-based calculator at https://jepusto.shinyapps.io/scdhlm, accessed on 23 September 2025; Pustejovsky et al., 2024). Visual analysis is the recommended method for understanding the functional relation of single-case designs (Ledford & Gast, 2018). Supplementing visual analysis with effect sizes is recommended to assist with reviews that pool data (e.g., meta-analysis). The between-case standardized mean difference effect size captures the magnitude of effects, while visual analysis focuses on levels, trends, and variability of data.

## 3. Results

**Figure 1:**
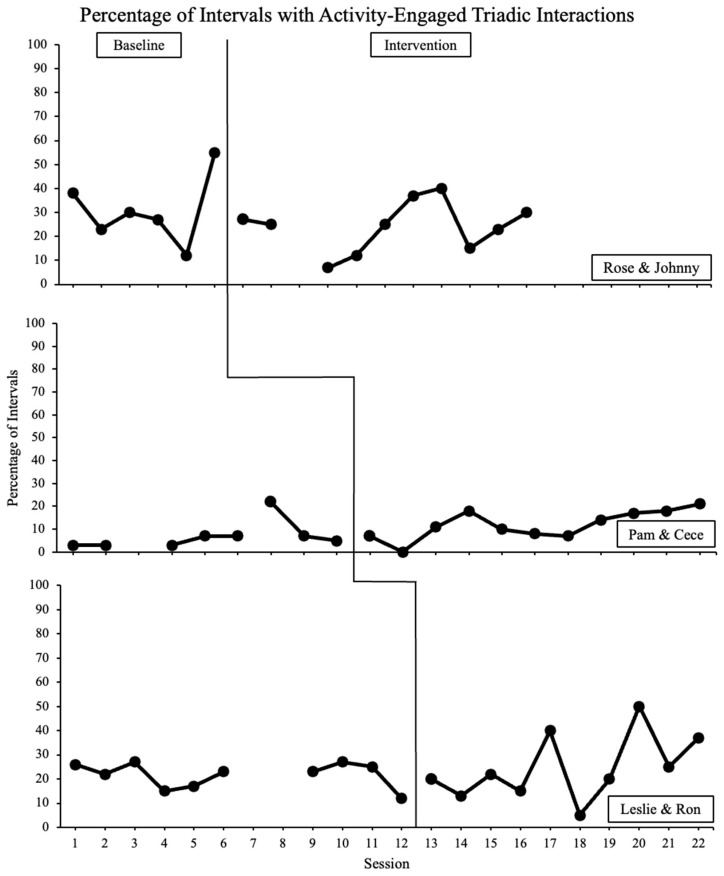
Graphical representation of data.

The current secondary analysis (see Ousley et al., 2022, for the original study) evaluated the collateral effects of a parent-implemented NDBI with an original focus on increasing child communication on activity-engaged triadic interactions between mother–child dyads during play routines in the home. Secondarily, we documented the means and ranges of other engagement levels throughout the study (i.e., unengaged, onlooker, object engaged, person engaged). For activity-engaged triadic interactions, the percentage of intervals in intervention remained roughly equivalent to baseline interactions. See Figure 1 for a graphical representation of the percentage of intervals, with triadic interactions being three mother–child dyads. For the other engagement levels, there was comparable consistency across phases. See Table 3 for means and ranges of all engagement levels in each phase for each mother–child dyad.

### 3.1. Overall Design-Level Outcomes

#### Activity-Based Triadic Interactions

At the design level, visual inspection of the graphs (i.e., visual analysis) and between-subjects standardized mean difference calculations were conducted. Visual analysis indicates no clear demonstrations of effects for activity-based triadic interactions. In other words, there is no functional relation as there is no clear change in level, trend, or variability for any of the three dyads. To supplement visual analysis, a between-subjects standardized mean difference calculation from Phase A (baseline) to Phase B (intervention) indicates a null to very small positive effect with an effect size of 0.061, SE = 0.218, 95% CI [−0.433, 0.555].

### 3.2. Individual Child Outcomes

#### 3.2.1. Johnny

Upon visual inspection of Johnny’s data on activity-engaged triadic interactions, there were varied levels in baseline and intervention, with no clear demonstration of effect and consistent overlap of data between phases. In baseline, Johnny displayed variable levels, with a potentially downward trend (the final data point in baseline may have been an outlier). Upon intervention, there was no clear change in level or variability. There was no clear indication of a trend in intervention, with all data overlapping with baseline levels. For individual levels of engagement, the average percentage of intervals spent in unengaged or onlooker engagement levels remained roughly the same between baseline and intervention. The percentage of intervals with object engagement as the highest level of engagement increased from 51% to 68% of intervals in the intervention compared to baseline. Interestingly, the mean percentage for person engaged decreased from 11% to 4% and activity-engaged triadic interactions decreased from 31% to 24% of intervals between baseline and intervention.

#### 3.2.2. Cece

Visual inspection of Cece’s data for activity-engaged triadic interactions reveals low levels with minimal variability in baseline. After intervention, levels were maintained, with much of the data overlapping with baseline levels. Average engagement levels were varied between baseline and intervention. Onlooking levels maintained the same (0%) between phases. Two engagement levels increased from baseline to intervention: person engaged (15% to 26%) and activity-engaged triadic interactions (7% to 12%). Two engagement areas decreased between baseline to intervention: unengaged (9% to 6%) and object engaged (69% to 56%).

#### 3.2.3. Ron

Graphical data of Ron’s levels of activity-engaged triadic interactions demonstrated low levels with mild variability in baseline. Upon intervention, Ron’s levels were generally maintained, with a slight increasing trend toward the end of intervention and one outlier (Session 18), as well as moderate levels of variability and significant overlap between phases. Overall, Ron maintained similar averages in onlooking (1%), object engagement (72%), and person engagement (2%) from baseline to intervention. Ron demonstrated a minor decrease in the average percentage of intervals unengaged (2% to 1%) and a slight increase in activity-engaged triadic interactions (22% to 25%).

## 4. Discussion

The current secondary analysis evaluated whether a brief parent-implemented NDBI, initially focused on increasing child communication and parent strategy use (Ousley et al., 2022), produced collateral effects on activity-engaged triadic interactions between mother–child dyads during play routines. Despite parents using all five strategies consistently and demonstrations of increases in child communication (Ousley et al., 2022), no clear functional relation emerged on activity-engaged triadic interactions (i.e., child–mother–child or mother–child–mother communicative exchanges while simultaneously remaining engaged in a shared activity). Visual inspection and effect size calculations showed minimal changes (null to very minimal positive effects), with consistently overlapping data from baseline to intervention for all three participants. While these data may initially suggest the intervention “didn’t work” for activity-engaged triadic interactions, they instead highlight more nuanced insights into interventions, skill complexity, and theoretical implications for skill development in young children with autism and developmental disabilities.

### 4.1. Language and Communication Interventions for Young Children with Autism and Developmental Disabilities

Many early interventions, including those that are implemented by parents, require multiple strategies and balancing strategy use by following the child’s lead (Ousley et al., 2024, 2025; Schreibman et al., 2015). Research has demonstrated that despite this complexity, parents can implement strategies with high levels of fidelity, especially when follow-up coaching is provided (Meadan et al., 2016). However, research typically focuses on one child outcome measure, which may not capture the variety or complexity of skills being targeted. Therefore, many researchers have opted to conduct secondary analyses of data to understand the secondary skills an intervention may impact (Bhana Lopez et al., 2024). The current evaluation included a parent-implemented intervention that was reduced to be brief and simplistic (only five strategies), with the goal of it being practically relevant and accessible to families and practitioners. However, despite the parents’ accurate and significantly increased implementation of strategies paired with promising increases in child communication (Ousley et al., 2022), gains in more complex social communication skills were not reflected in this secondary analysis.

The brevity of intervention (4–6 weeks) likely limited its impact on building more complex skills that require both parties responding to the intervention, like activity-engaged triadic interaction. While parents reported that the intervention was highly acceptable, impactful, and that they would continue using the intervention past the brief intervention (see Ousley et al., 2022), this positive perception does not eliminate the difficulty of balancing the implementation of strategies with centering and responding to the child on a moment-by-moment basis. It is likely that these more complex skills take longer to master (Vernon et al., 2012), as the need to monitor the child’s response, implement multiple strategies (e.g., modeling language, rewarding and expanding language), and string strategies together in real time may be challenging for parents who are not intervention specialists.

This discussion highlights a tension in parent-implemented interventions: although parents can learn and use multiple strategies, balancing strategy use with responsiveness and “in-the-moment” decision-making may require more time to master from the parents’ perspective (i.e., balancing strategies and responding based on their child’s response) and child experience (i.e., many foundational skills are required for more complicated skills). It is possible that these complex skills may require a targeted, explicit approach or more coaching and practice with building their skills (both the parent and child) before seeing behavioral outcome results.

### 4.2. Skill Complexity

Developmental milestones can often be broken down into foundational subskills that are critical to building more complex skills. Typically, with interventions, children gain these skills in a similar and predictable order, each building upon the other and becoming more complex (e.g., focusing on joint attention prior to joint engagement prior to triadic interactions; Bakeman & Adamson, 1984). With the current intervention, increases in child communication were observed, but these increases did not translate directly to activity-engaged triadic interactions, potentially reflecting the incremental nature of skill acquisition. Important to note, an additional empirical evaluation, such as isolating joint engagement outcomes and triadic interactions, is required to empirically support this theoretical implication.

Additionally, child-level factors (e.g., communication delays, varied play activities) may encourage a slower emergence of more complex behaviors. For example, it may be that activity-engaged triadic interactions may need additional coaching or exposure before a clear change in behavior is developed, as immediate behavioral changes are not apparent in the current study. There is evidence to suggest that prior to demonstrating skills through behavior, individuals with disabilities demonstrate these skills through other markers. Eye-tracking has been shown to provide an objective, sensitive measure of behavioral change in response to intervention (Fletcher-Watson & Hampton, 2018). Neuroimaging reveals that the development of complex attentional skills in children may be observed in EEG scans prior to behavioral changes (Pietto et al., 2018). Eye-tracking and EEG have been used in combination to evaluate the efficacy of social and attentional interventions in autistic children (Wall et al., 2025). These present promising methods to evaluate interventions alongside behavioral measures, to better understand processes behind the development of activity-engaged triadic interactions. Incorporating such tools in future research could provide insight into how complex skills are built and the timing of observable behavioral change.

### 4.3. Practical Applicability of Brief, Intensive Research-Based Interventions

Our findings highlight important considerations for the practical application of interventions and the use of measurement systems. In the original study (Ousley et al., 2022), clear gains were observed in parent implementation of strategies, thus leading to increases in child communication. However, when evaluating the more complex behavior of activity-engaged triadic interactions, which combined both parent and child behavior into one dependent variable, clear gains were not observed. This may underscore the importance of aligning interventions with specific, observable targets. Further, this highlights a theoretical implication that broader, multi-step skills may require prolonged or more focused efforts to emerge.

There are implications for both practitioners and researchers. When practitioners are not seeing expected changes in child outcomes, practitioners should reevaluate the skill being measured and the intervention being implemented to ensure that the measurement system is capturing child behavioral changes (e.g., increases in communication, see Ousley et al., 2022). It may be that progress in foundational skills (e.g., frequency of communication) may precede visible changes in complex outcomes (e.g., triadic interactions). Further, practitioners need to ensure their measurement systems and intervention targets align with one another and are sensitive enough to capture both subtle early signs for skill emergence and fully developed complex developmental milestones (Raulston et al., 2024). Researchers should conduct secondary analyses that extend the primary focus of the intervention of existing single-case experimental design datasets to identify additional intervention outcomes, including those that may yield null effects, to build a comprehensive understanding of specific interventions. Having a more well-rounded understanding of the impact of various interventions, particularly those that are attempting to be feasible for practical applications, will provide data for practitioners to consider when selecting interventions to target a skill appropriate for their client. This information can provide valuable insight into skill trajectories, “active ingredients” of interventions, and areas to refine training and coaching approaches.

### 4.4. Limitations and Future Research

Like all research, several limitations warrant attention. First, there was a small sample size (i.e., three dyads) and a varied population within this intervention (i.e., three different disabilities). Additionally, all parents were mothers. This limits the generalizability of our results. Future research should include larger sample sizes and replicate the current coding scheme across other NDBIs to better understand the generalizability of NDBIs on more complex skills, such as activity-engaged triadic interactions. Relatedly, the play sessions varied between and within cases to enhance the social and ecological validity of the study. In other words, the children played with different toys and activities from one another and from one session to another. This further limits the generalizability of the results. Future research may wish to standardize play across dyads to prevent the possible variance from alternating play activities. Second, the rising baseline data for Johnny poses a threat to internal validity. This high variability may have been due to natural fluctuation; however, without additional probes, it is unclear what may have caused the variable baseline. If the current dependent variable were the primary dependent variable that was informing the response-guided approach, additional data would have been collected to evaluate if the data point was an outlier or part of a change in outcome. Third, the intervention was very brief (4–6 weeks), which may have been a barrier to success with more complex skills. Future research should evaluate the effects that brief and extensive coaching packages have on the development of complex skills.

Finally, and perhaps most importantly, we suggest that these data be interpreted with extreme caution. Our coding system did not separate the two primary areas of our target dependent variable: joint engagement and triadic interactions. Further, we did not code partial activity-engaged triadic interactions (e.g., dyadic interactions while being jointly engaged in an activity). Because this study did not directly break apart which skills may have emerged as a result of the intervention, any discussion regarding joint engagement or triadic interactions as precursors to more complex skills remains theoretical rather than empirically supported by these data alone. To empirically support this claim with methodological rigor, future research should break down more complex social communication skills into their subskills. Such an approach would allow for the detection of subtle but meaningful behavioral changes, while simultaneously providing practical implications through empirically supported data.

## 5. Conclusions

In summary, this secondary analysis provides insight into the nuances around building complex developmental skills in young children with autism and other developmental disabilities through brief parent-implemented NDBIs. While the original aims of the intervention were met (i.e., increases in parent strategy use and child communication), there was no clear collateral effect on how well the parents and children were able to string together their skills, as measured through activity-engaged triadic interactions, within the 4–6 week coaching period. This highlights a critical challenge with interventions that target developmental skills in young children with autism and developmental conditions: the balancing of strategy use with responsive, in-the-moment reactions. For expert practitioners, this may be feasible. However, for natural change agents, such as parents, this may be more challenging to navigate. Researchers should evaluate secondary effects their interventions may have on related or more complex developmental skills, while breaking down the complex skill into subskills, even when the results are null, to help provide a more robust understanding of what interventions (e.g., brief, simple, complex) can yield what developmental skills (e.g., communication, joint attention, triadic interactions) to assist practitioners in selecting and implementing effective yet feasible interventions in their practice. While brief parent-implemented interventions can increase the frequency of child communication (Ousley et al., 2022), null effects were found when evaluating a more complex social communication skill. These findings highlight the need for future research to break apart complex skills into subskills to detect any subtle changes in child outcomes.

## Figures and Tables

**Table 1:** Participant demographics and assessment results.

			VABS-3		
Participant	Age	Child Diagnosis	ABC Score	Communication Standard Score	Socialization Standard Score
Dyad 1					
Johnny	5 years 2 months	Autism Spectrum Disorder	71 (*SD* = −2)	56 (*SD* = −3)	76 (*SD* = −1.5)
Rose	39 years				
Dyad 2					
Cece	2 years 2 months	Williams syndrome	68 (*SD* = −2)	58 (*SD* = −3)	90 (*SD* = −0.5)
Pam	32 years				
Dyad 3					
Ron	2 years 0 months	Developmental delay (communication)	93 (*SD* = −0.5)	85 (*SD* = −1)	98 (*SD* = 0)
Leslie	29 years				

*Note.* Dyads are listed by child first, then mother. VABS-3 = Vineland Adaptive Behavior Scale—Third Edition; ABC = Adaptive Behavior Composite Score.

**Table 2:** Operational definitions and ranking of engagement levels.

Level of Engagement	Definition	Example
0. Unengaged	Child is not focused on or interacting with their mother nor any objects, activities, or toys.	Child is swaying back and forth with no apparent focus on any item, object, person, or activity.
1. Onlooker	Child is watching their mother or toy/object but is not actively involved in interacting with the mother or the activity.	Child is standing a few feet away from their mother, watching them read a book, without showing any interest in the mother or the book.
2. Object Engaged	Child is playing exclusively with an object with no social behavior with the mother.	Child is holding a toy truck and pushing it down a car ramp. The child is not showing any interaction or conversing with their mother.
3. Person Engaged	Child is focused on and interacting with their mother with no object involved.	Child is looking at their mother, jumping up and down, and the mother continues singing a song.
4. Activity-Engaged Triadic Interaction	Child interacting with their mother and a shared object or activity at the same time (i.e., joint engagement) *plus* a back-and-forth triadic interaction between both parties (child–mother–child or mother–child–mother exchanges).	The child goes up a slide while saying “up.” The parent responds with “you did it” while repositioning herself to be at the bottom of the slide. Child says “I did” following their mother’s comment.

*Note.* Definitions for onlooker, object engaged, and person engaged were developed based upon Joint Attention, Symbolic Play, Engagement, and Regulation (JASPER; Kasari et al., 2021).

**Table 3:** Means and ranges of the percentage of intervals for each engagement state.

	Child Engagement/Interaction Level				
	Unengaged	Onlooking	Object Engaged	Person Engaged	Activity-Engaged Triadic Interaction
Johnny (Dyad 1)					
Baseline					
*M*	3%	2%	51%	11%	31%
(range)	(0–10%)	(0–10%)	(2–88%)	(0–58%)	(12–55%)
Intervention					
*M*	4%	1%	68%	4%	24%
(range)	(0–8%)	(0–7%)	(43–85%)	(0–18%)	(7–40%)
Cece (Dyad 2)					
Baseline					
*M*	9%	0%	69%	15%	7%
(range)	(0–22%)	(0–2%)	(53–87%)	(7–22%)	(3–22%)
Intervention					
*M*	6%	0%	56%	26%	12%
(range)	(0–15%)	(0–2%)	(38–80%)	(2–45%)	(0–21%)
Ron (Dyad 3)					
Baseline					
*M*	2%	1%	72%	2%	22%
(range)	(0–10%)	(0–3%)	(60–85%)	(0–8%)	(12–27%)
Intervention					
*M*	1%	1%	72%	2%	25%
(range)	(0–5%)	(0–3%)	(50–88%)	(0–3%)	(5–50%)

## Data Availability

De-identified raw data (i.e., engagement level for each dyad for each session) and graphs represented in this study are attached as Supplemental Materials.

## Supplementary Materials

The following supporting information can be downloaded at: https://www.mdpi.com/article/10.3390/bs16010147/s1, Deidentified raw data and graphs.
